# Bi-directional Scan Pattern Effects on Residual Stresses and Distortion in As-built Nitinol Parts: A Trend Analysis Simulation Study

**DOI:** 10.1007/s40192-023-00292-9

**Published:** 2023-01-30

**Authors:** Medad C. C. Monu, Yalda Afkham, Josiah C. Chekotu, Emmanuel J. Ekoi, Hengfeng Gu, Chong Teng, Jon Ginn, Jennifer Gaughran, Dermot Brabazon

**Affiliations:** 1 I-Form Advanced Manufacturing Research Center, Advanced Processing Technology Research Center, Dublin City University, Dublin, Ireland; 2 School of Physics, School of Mechanical and Manufacturing Engineering, Dublin City University, Dublin, Ireland; 3 Ansys, Inc., 1441 West Ute Blvd, Suite #335, Park City, UT 84098 USA

**Keywords:** Part-scale simulation, Scan pattern, In situ monitoring, Residual stress, Nitinol, Inherent strain

## Abstract

In this paper, a part-scale simulation study on the effects of bi-directional scanning patterns (BDSP) on residual stress and distortion formation in additively manufactured Nitinol (NiTi) parts is presented. The additive manufacturing technique of focus is powder bed fusion using a laser beam (PBF-LB), and simulation was performed using Ansys Additive Print software. The numerical approach adopted in the simulation was based on the isotropic inherent strain model, due to prohibitive material property requirements and computational limitations of full-fledged part-scale 3D thermomechanical finite element approaches. In this work, reconstructed 2D and 3D thermograms (heat maps) from in situ melt pool thermal radiation data, the predicted residual stresses, and distortions from the simulation study were correlated for PBF-LB processed NiTi samples using selected BDSPs. The distortion and residual stress distribution were found to vary greatly between BDSPs with no laser scan vector rotations per new layer, whereas negligible variations were observed for BDSPs with laser scan vector rotations per new layer. The striking similarities between the reconstructed thermograms of the first few layers and the simulated stress contours of the first lumped layer provide a practical understanding of the temperature gradient mechanism of residual stress formation in PBF-LB processed NiTi. This study provides a qualitative, yet practical insight towards understanding the trends of formation and evolution of residual stress and distortion, due to scanning patterns.

## Introduction

**Fig. 1:**
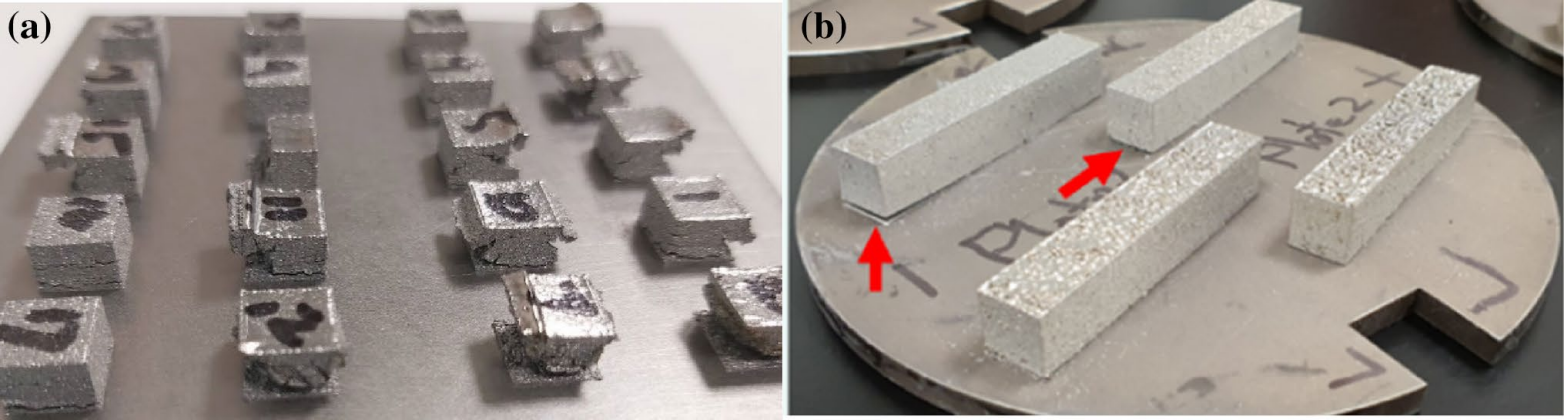
Warpage distortions in PBF-LB processed NiTi **a** cuboids (7 mm × 7 mm × 7 mm) and **b** rectangular bars (55 mm × 10 mm × 10 mm)

One of the most common metal additive manufacturing (AM) processes is powder bed fusion using a laser beam (PBF-LB), also known as selective laser melting. In this process, a high-powered laser beam is scanned over a layer of powder, melting, and enabling the powder to solidify into the required shape as it moves. Layer by layer, more powder is added, and the laser beam irradiates these powders to form 2D layers that metallurgically bond to the previous layer until the desired component height is reached [1, 2]. In this technique, an inert chamber atmosphere is used to prevent oxidation and the process is ideal for producing small-to-medium-sized and precise components. During this process, laser beams generate significant thermal gradients, which cause residual stresses to form. These residual stresses may cause deformation (i.e. distortions) in the as-built parts. Distortion occurs when a small amount of material is heated to its melting point on a much colder previous layer and build substrate. The cooling of the material causes a thermal contraction at the top of the part as the molten material solidifies, stretching the lower portions of the part upwards. This cyclic expansion and contraction induce higher stresses, which can cause the part to yield, resulting in permanent deformation [3, 4]. Figure 1 shows pictures of (a) a failed build of 7 mm Nitinol (NiTi) cuboids due to extreme warpage, and (b) small-scale distortions in some NiTi rectangular bars, produced by the co-authors, which effectively rendered the bars unacceptable for the intended application due to the required stringent dimensional tolerances.

Distortion during metal additive manufacturing can be mitigated by preheating build plates, continuous heating of the build chamber during manufacture [5, 6], powder pre-heating [6], laser scanning strategy adopted [7], and restraints [8]. Despite obvious benefits, these distortion mitigation strategies are limited in their ability to reduce overall part distortion and residual stresses in the powder bed fusion (PBF) processes. As a result, bending forces may not be fully dissipated and residual stresses will remain where physical part distortion may not be visible.

Laser scan strategy has been identified as a critical factor that influences mechanical properties [9, 10], final microstructure [11–14], amount of locked-in residual stresses [7, 10, 15, 16] and the ensuing levels of distortion in as-built parts [17–20]. In all afore-cited works, the most direct method of evaluating residual stresses and distortion is by expensive and time-consuming experimental measurements. This further creates material wastage and drives up the overall cost of production or research. However, numerical simulation offers a more cost-effective alternative and can be applied in the design phase to achieve rapid evaluation [21–23]. The simulation-based analysis provides information for users to identify residual stress distribution and distortion during a product design stage. Thereafter, users can re-design the geometry and/or optimize process parameters for the final geometry/part whilst simultaneously minimizing build failure rates [24, 25]. Larry et al. [26] adopted a coupled thermomechanical finite element analysis for investigating the effect of unidirectional and alternating (meander) laser scan strategies on the generation of residual stress in PBF-LB processed Ti–6Al–4V parts. Residual stress distribution and plastic strain varied between the two laser scan strategies due to differences in the thermal history. However, the two laser scan strategies generated residual stress magnitudes that were not substantially different. The most important recommendation from the findings from the work by Larry et al. [26] is that laser scan strategies with predominant long scan vector lengths should be avoided and the direction of scan vectors should be orientated uniformly, to produce isotropic stress fields in the as-built part. Also by coupled thermomechanical finite element analysis, Cheng et al. [27] investigated the effect of different laser scanning strategies on the temperature, residual stress and deformation of an 8 × 8 × 1 mm IN718 part of only 3 layers. Findings from the study show that for out-in and horizontal line strategies, maximum stresses occur along with the *X-* and *Y*-directions. However, a 45° inclined line-scanning strategy was observed to reduce these residual stresses in both directions. Furthermore, the locations of *X* and *Y* directional stress concentrations were observed to be at the edge of the deposited layers and the interface between the deposited layers and the substrate for all cases. Zaeh and Branner [28] developed a global 3D numerical model that can substitute the scanning vectors of layers with combined scanning areas, so that part-level thermomechanical simulations may be performed. Other works based on 3D thermomechanical FEA can be found in Ref. [29–32], and the review study by Teng et al. [33].

Even though the cost of running multiple experiments and material wastage is drastically reduced, the main drawbacks of adopting thermomechanical FEA include (1) computational time, (2) exponential computational costs, and (3) limited computational power [34]. For complex metal parts that may contain thousands of layers with numerous scan tracks and varying scan vector lengths, part-scale computational study on residual stresses and distortion by detailed thermomechanical simulation is prohibitive. Another prohibitive factor in the use of 3D thermomechanical FEA models for predicting residual stresses and distortion in PBF-LB is the sheer amount of material property information required. This is particularly challenging for nickel–titanium alloy systems (Nitinol), but readily available for other metal alloy systems like Ti–6Al–4V, and 316L stainless steel.

### The Unique Case of Nitinol (NiTi)

A full-fledged part-scale 3D thermomechanical FE modelling of residual stresses and distortion for PBF-LB NiTi will require immense temperature-dependent properties according to governing equations in Eqs. (1–5) [35].

1 $$\rho {C}_{p}\frac{\text{d}T}{\text{d}t}=\frac{\delta }{\delta x}\left(k\frac{\delta T}{\delta x}\right)+\frac{\delta }{\delta y}\left(k\frac{\delta T}{\delta y}\right)+\frac{\delta }{\delta z}\left(k\frac{\delta T}{\delta z}\right)+Q$$

2 $$\text{Heat loss due to radiation:}\,\, -k\nabla T.{\varvec{n}}=\sigma \zeta \left({T}^{4}-{T}_{0}^{4}\right)$$

3 $$\text{Governing equation for mechanical analysis:}\,\, \nabla \sigma +\rho b=0$$

4 $${\varepsilon }_{\text{total}}={\varepsilon }_{\text{elastic}}+{\varepsilon }_{\text{plastic}}+{\varepsilon }_{\text{thermal}}$$

5 $${\varepsilon }_{\text{thermal}}=\alpha \Delta T$$

where* Q* represents the volumetric heat input term, *t* is time, *σ* is the Stephan–Boltzmann constant in Eq. [2], the stress tensor in Eq. [3], *ε* is strain, and *b* is the body force per unit volume [35]. Specifically, on the temperature-dependent property terms, *ρ* is the density (g/cm^3^), thermal diffusivity is *α* [m^2^ s^−1^], *Cp* is the specific heat capacity [J kg^−1^ K^−1^], *k* is the thermal conductivity [W m^−1^ K^−1^], and *ζ* is the emissivity. These properties are required at temperatures, *T* ranging from 20 °C to at least 1700 °C. (Melting temperature of NiTi is 1310 °C.) Nitinol is a nearly stoichiometrically equal alloy of pure Ni and pure Ti, and these temperature-dependent properties are difficult to obtain due to solid-state thermal gradient-induced phase changes. The difficulty in collating such data is exasperated by the fact that there is approximately a 20 °C drop in the transformation temperature per 0.1% change in Ni to Ti ratio above 50% Ni [36, 37]. This sensitivity to transformation temperature results in huge changes in the temperature at which shape recovery occurs for the finished part/component since the temperature of the martensitic transition of NiTi is essentially dependent on the chemical composition [27]. More so, the extreme melt pool temperatures (peak temperatures) due to laser irradiation [15], processing chamber pressures of > 50 mbar above ambient atmosphere [38], extreme cooling gradients larger than 10^7^ K/m and cooling rates of 10^7^ K/s considering a typical laser scan speed of the order of 1 m/s [39, 40] during any PBF-LB process further complicate the acquisition of such temperature-dependent properties and model prediction accuracy. More so, the minimum purging inert gas flow rate of 15 l/min [38] inadvertently contributes to the high cooling rates during the build process.

There is approximately an 18.5% difference between the boiling points of pure nickel (2730 °C) and that of pure titanium (3287 °C). Unsurprisingly, it is well-reported in the published literature that nickel vapourizes more readily than titanium during the inherently extreme PBF-LB processing temperatures [41–43]. This effectively changes the atomic percentage of Ni in the final PBF-LB processed NiTi part when compared to the NiTi powder feedstock. The consequences of such changes include enormous differences in the martensite and austenite phase transformation temperatures, the extent of phases (martensite and austenite) present, and the ensuing changes from the targeted or desired superelasticity and shape memory behaviour. Unfortunately, the reported variations in the extent of Ni evaporation, precipitates such as Ni_4_Ti_3,_ the unavailability of Gibbs free energy function for precipitates like Ni_4_Ti_3_ [44, 45], and the sheer complexity of the PBF-LB processing conditions such as purging gas type, flow rate, powder emissivity, chamber pressure, and oxygen levels make CALPHAD-based property calculations for NiTi challenging. At standard temperature and pressure (STP), there is also a huge disparity in the existing literature, for the thermal conductivity (from 1.8 to 28 W/m K), and specific heat capacity (from 320 to 3300 J/kg K) of NiTi alloy [46]. The composition-temperature-dependent-property sensitivity of Nitinol means that the few temperature-dependent properties of cast Nitinol, virgin Nitinol powder, or CALPHAD-based property predictions do not serve as good substitutes for use in a full-fledged 3D thermomechanical modelling of PBF-LB processed Nitinol (NiTi).

**Fig. 2:**
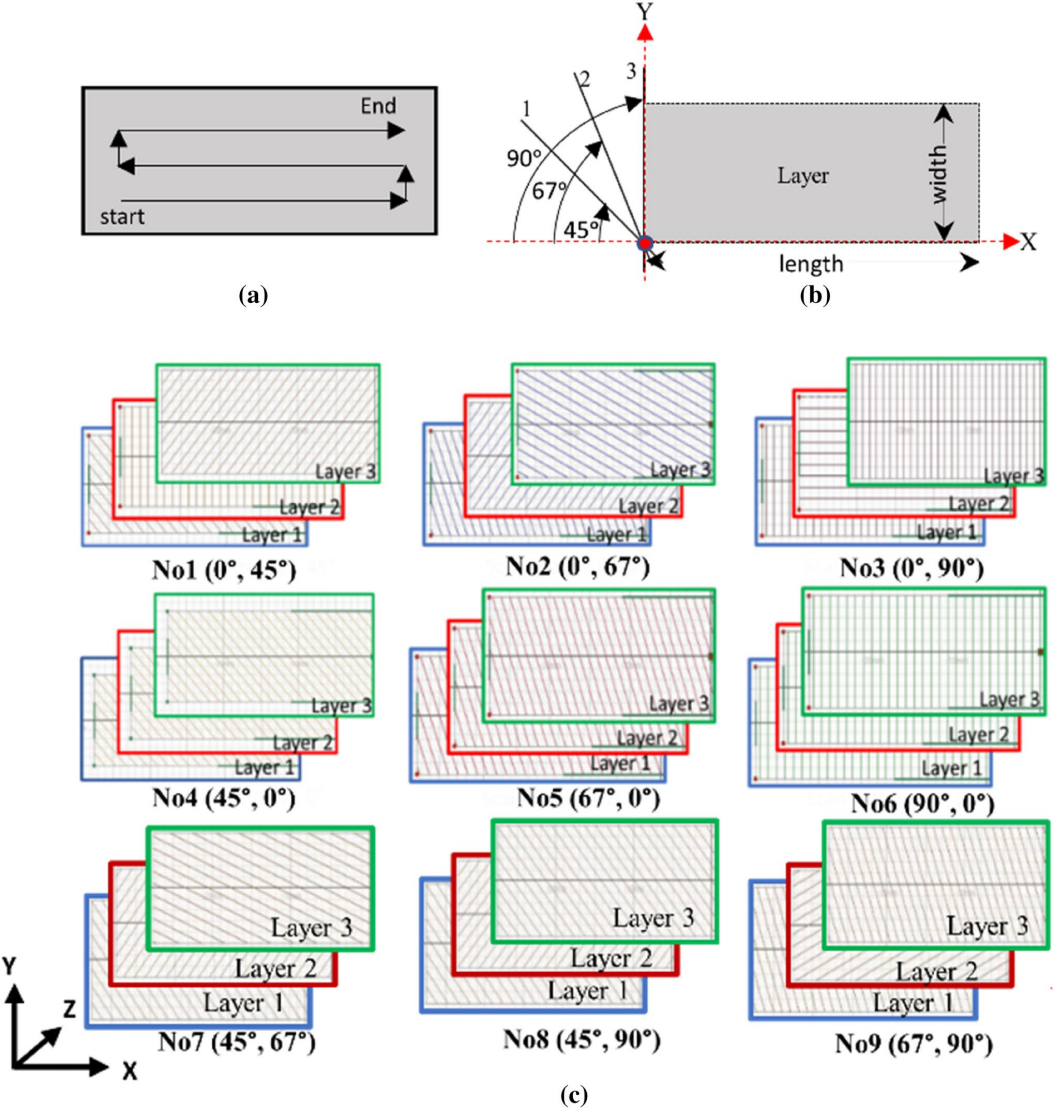
**a** Schematic illustration of bi-directional scan strategy, **b** layer slicing coordinate system, where lines 1, 2 and 3 represent the angular references for hatch lines of either 45°, 67°, or 90° laser scan vector orientations, respectively, and **c** bi-directional scan patterns (BDSP) with different starting layer angle and layer rotation angle combinations using 0°, 45°, 67°, and 90° degrees. Note that No. 1 (0°, 45°) refers to the first BDSP with a 0° starting layer laser scan vector angle and 45°-laser scan vector rotation angle per new layer, and this applies to all

Furthermore, the existing literature suggests that the bi-directional scan strategy shown in Fig. 2a is not well suited for the PBF-LB processing of NiTi, as it introduces large residual stresses and distortion in the part [27, 37]. In the absence of the temperature-dependent properties of NiTi, the development of part-scale (macroscale) PBF-LB simulations or models for NiTi based on a 3D thermomechanical numerical/FE model that accounts for laser scan vector effects is challenging. The main error sources of a 3D thermomechanical numerical/FE model include uncertainty in material properties, the agglomeration strategy in the model, and prescribed boundary conditions necessary for computational expediency [31]. For such scenarios, the inherent strain-based scan pattern simulation within the Ansys Additive Print® software suite enables the trend analysis of residual stresses and distortions using only the materials’ room temperature mechanical properties, a user-inputted strain scaling factor (SSF) and anisotropic strain coefficients (ASC).

One limitation of Ansys Additive Print's inherent strain-based scan pattern simulation is that it may result in either over or underprediction of residual stresses and distortion magnitudes in PBF-LB parts, regardless of whether *J*_2_ plasticity or linear elastic stress mode is used. *J*_2_ plasticity is a plasticity theory (yield criterion) which suggests that the second deviatoric stress invariant, *J*_2_ $\left\{=\frac{1}{6} \left[{\left({\sigma }_{1}-{\sigma }_{2}\right)}^{2}+{\left({\sigma }_{1}-{\sigma }_{3}\right)}^{2}+{\left({\sigma }_{2}-{\sigma }_{3}\right)}^{2}\right]\right\}$ must reach a critical value beyond which, a material plastically deforms (i.e. yields) [47]. ${\sigma }_{1}, \,{\sigma }_{2},\, \text{and} \,{\sigma }_{3}$ represent the principal stresses [47].

The possible sources of the mismatched predicted values (i.e. percentage error between simulated and experimental values) are the temperature-dependent material properties, flow softening, and microstructure evolution is neglected in the simulation model. Notwithstanding, the general trend (location and/or distribution) remains unchanged [23, 34, 35]. Despite this limitation and the associated difficulty of predicting absolute values, such models can be used to determine the trend of stress/strain change between input conditions. In comparison with the 3D thermomechanical numerical/FE-based models, the “Inherent or assumed strain-based” numerical technique provides a middle ground between detailed quantification and qualification of predicted residual stress and distortion. This provides an effective tool for trend analysis of desired PBF-LB components with superior computing speeds, quicker geometry assessment during the design stage, and requires fewer material properties required to solve the model. Thus, this work is a trend analysis of the effects of different combinations of scan vector orientations based on the bi-directional scan strategy, for the PBF-LB processing of bars made from NiTi alloy, using the Ansys Additive Print^®^ 2021 software suite.

## Methodology

### Scan Pattern Simulation

The three major types of strain modes relevant for PBF-LB simulation in Ansys Additive Print are assumed strain simulation (ASS), scan pattern simulation (SPS), and thermal strain simulation (TSS). An advantage of the Ansys Additive Print package is that it uses a layer-based isotropic inherent strain approach to predict residual stresses and distortions. Instead of simulating each laser pass for each physical layer, a simulated layer that contains all laser passes per the full dimension of a layer is lumped during the simulation. This layer-based feature enables for investigation of residual stress evolution as build height increases. For simulation purposes, the thickness of the lumped layers is defined by the voxel size and voxel sample rate, details of which will be provided in “Voxelization” section. This study utilizes the scan pattern simulation (SPS) mode, since using the thermal strain simulation (TSS) will also require temperature-dependent properties as with a full-fledged part-scale 3D thermomechanical FE modelling.

This SPS mode uses the same average strain magnitude from ASS but subdivides that strain into anisotropic components based on the local orientation of scan vectors within the part by applying a set of three anisotropic scaling coefficients (ASC), ASC_||_, ASC_⊥_ and ASC_Z_. ASC_||_ and ASC_⊥_ are used to scale the assumed strain into two in-plane strains which are in parallel and transverse directions to the laser scan direction within each layer of a scan. ASC_Z_ is used to scale the assumed strain in the vertical build direction *Z*. The assumed strain (also known as inherent strain) simulation mode is the fastest simulation type available and assumes that within a part that is being built by PBF-LB, a constant, isotropic strain occurs at every location throughout the part/object. This isotropic assumed strain is mathematically given as:

6 $$\text{Isotropic assumed strain}=\text{SSF}\frac{{\sigma }_{\text{yield}}}{E}$$

where ${\sigma }_{\text{yield}}$ and $E$ represent the material yield strength (MPa) and elastic modulus (GPa) [48], and SSF represents the strain scaling factor, which is used as a calibration scaling coefficient. Similarly, the anisotropic inherent strain represents *x-*, *y-*, and *z*-directions:

7 $$ {\text{Anisotropic inherent strain}}\,\,||{\text{ = ASC}}_{||} \times \,{\text{isotropic assumed inherent strain}} $$

8 $$ {\text{Anisotropic inherent strain}}_{\bot} = {\text{ASC}}_{ \bot } \times {\text{isotropic assumed inherent strain}} $$

9 $$ {\text{Anisotropic inherent strain}}\; \, Z = {\text{ASC}}_{Z} \times {\text{isotropic assumed inherent strain}} $$

The SSF and ASCs quantify the variables unique to each build scenario, and it is a set of calibration factors that are experimentally determined for each machine/material/strain/stress mode combination of interest. For example, SSF of 1 implies that the ASS outputs/results are as is (not scaled) and are independent of the PBF-LB machine factors or scan pattern used. In contrast, a calibration step to determine SSF of more than or less than 1 will have a multiplicative scaling effect on the ASS output as indicated in Eq. (1). This approximates the combined effects of the PBF-LB parameters, machine factors, and stress mode (linear elastic or *J*_2_-plasticity) [49]. Both parameters are geometry independent, and further details on the calibration procedure are provided in Ansys Additive Print documentation [48]. In this study, the effect of the local orientation of the scan vectors using the SPS mode, is implemented as bi-directional scanning strategy (see Fig. 2a). This bi-directional scanning strategy is defined by nine combinations of laser scan vector angle for the first layer (i.e., start layer angle), and laser scan vector angular rotations per new layer (i.e., layer rotation angles) of 0°, 45°, 67°, and 90° based on the slicing coordinate system illustrated in Fig. 2b. The resulting bi-directional scan patterns are illustrated in Fig. 2c. Note that the bi-directional scan pattern is hereafter abbreviated as BDSP and adopted for the rest of this study.

As with Eqs. (7–9), the three ASCs have a multiplicative scaling effect on the calculated inherent strain values within each voxel, to give location-dependent stress/strains due to these different scan vector angular orientations. Using the SPS module, layer thickness, scan type, and the already discussed anisotropic strain coefficients are the only required simulation input parameters. Detailed model set-up parameters are shown in Table 1. It is worth noting that a detailed calibration of the ASC would be required to be able to predict the absolute stress values resulting from the process. However, due to the requirement for extensive and expensive testing, it was not possible to obtain the actual stress values from the various processing conditions necessary to achieve full calibration. Instead, to be able to examine via trend analysis, how the scanning patterns comparatively affect the strain, the selected ASC values were the software default settings.

**Table 1:** Examined model set-up parameters

Simulation mode	Scan pattern
Stress mode	Linear elastic
Strain scaling factor	1
Anisotropic Strain coefficients (||)	1.5
Anisotropic Strain coefficients (┴)	0.5
Anisotropic Strain coefficients (Z)	1
Starting layer angle	0°, 45°, 67°, 90°
Layer rotation angle	0°, 45°, 67°, 90°
Initial temperature (material and substrate)	20 °C

The notation used to describe the BDSPs in Fig. 2 comprises a scan number, starting layer angle (°), and layer rotation angle (°). For example, the fourth scan strategy No4 (45°, 0°) refers to the fourth (i.e. No4) BDSP characterized by 45° first or starting layer angle, and 0°-layer rotation angle. The starting layer angle (here 45°) is the angle the hatch lines (laser scan vectors) make with the *X*-axis, which also determines the angle at which the first layer hatch lines will be scanned. The layer rotation angle (here 0°) is the angle that determines how much the angle of the hatch line is changed layer by layer. By extension, this layer rotation angle determines the major laser scan vector orientation changes from layer to layer [49–51]. In Ansys Additive Print, these positive angles are measured in clockwise rotation from a reference point on the Cartesian *X*-axis, as illustrated in Fig. 2b. For the layer slicing coordinate system in Fig. 2b, lines 1, 2 and 3 represent the angular references for the hatch lines, needed to implement either 45°, 67°, or 90° laser scan vector orientations with respect to the *x*-axis, respectively. This is in line with the default implementation strategy of commercially available slicing software, such as Autodesk Netfabb, and the interpretation of sliced 3D models by commercially available PBF-LB equipment software suites, such as Aconity Studio®. The only exception is when a user specifies a negative angle, in which case, the angular measurement will be taken from an anti-clockwise direction. It is important to note that for BDSP No1, No2 and No3, which have a 0° starting layer angle command, the layer rotation angles override this command. This means that the first layer scan will be oriented by the exact value of the layer rotation angle, thereby neglecting the 0° starting layer angle command. Therefore, the schematics in Fig. 2a begin with 1st layer hatch lines oriented at 45°, 67°, or 90°, for BDSP No1, No2 and No3, respectively. However, if a BDSP with a 0° starting layer angle and the 0°-layer rotation angle is to be investigated, this would result in scan vectors (hatch lines) that are persistently parallel to the *X*-axis. This latter case is not included in the study but is noted here for an explanation of the software operation. Simulation data from the Ansys Additive Print software are exported and post-processed in ParaView [52], an open-source, multi-platform data analysis and visualization application.

### Voxelization

**Fig. 3:**
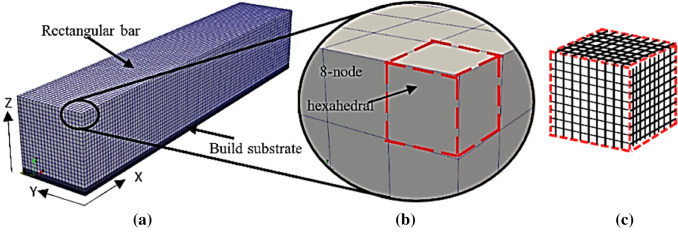
Simulation set-up **a** voxelated rectangular bar and build substrate, **b** regular 8-node quadratic hexahedral elements, and **c** illustration of a voxel divided into 1000 sub-voxels (i.e. voxel sample rate of 10 = 1000 sub-voxels)

The dimensions of the rectangular bar are 55 mm (length) $\times$ 10 mm (width) $\times$ 10 mm (height), as shown in Fig. 3a. The voxels of the bar are 8-noded regular quadratic hexahedral elements (i.e. a cuboid) measuring 500 µm (length) × 500 µm (width) × 500 µm (height) (see Fig. 3b). At 500 µm voxel height, this results in 20 lumped layers with a powder height resolution of 12.5 layers (i.e. 500 µm voxel height divided by 40 µm layer thickness). To improve the voxel representation and FE calculation accuracy for the bar (especially the corners/edges), the voxel sample rate (VSR) was set to 10. The VSR represents the cubic number of sub-voxels within each voxel. Thus, at 10 VSR, each voxel is subdivided into 1000 sub-voxels (10^3^) measuring 50 µm × 50 µm x 50 µm each (Fig. 3c). In this study, the rectangular bar and substrate are assumed and modelled with the same material (i.e. NiTi) properties and the thickness of the substrate is assumed to be infinitely thick. The bottom surface of the substrate is constrained in all directions.

### Material Properties

Tensile test specimens were fabricated from a Ni_50_Ti_50_ (at.%) alloy powder utilizing the AconityMINI® PBF-LB system. The processing conditions were set to 180 W laser power, 1200 mm/s scan speed, 70 µm hatch spacing, spot size was 50 µm, layer thickness was 40 µm, and the scan pattern was bi-directional as per simulation pattern No3 (0°, 90°), shown in Fig. 2c. Testing was carried out according to ASTM E8/E8M standard, using four (4) small size round specimens of length, 29 mm; diameter 4 mm and gauge length 10 mm. Tensile testing was performed to failure using a 50 kN Zwick/Roell testing system with a crosshead speed of 1 mm/min and a preload of 5 N in the specimen build direction. A Class B-1 axial extensometer (Epsilon Technology Corp) was utilized to record specimen elongation during testing. The resultant material properties used for the simulation are presented in Table 2.

**Table 2:** Average tensile test results for Ni_50_Ti_50_ martensitic printed via PBF-LB, *n* = 5

Property	Value
Yield strength	188.87 MPa
Elastic modulus	34.03 GPa
Poisson ratio	0.33

### Melt Pool Thermal Radiation Data Visualization

To understand the formation of residual stresses, the predicted residual stresses of a few layers were compared to the temperature distribution within a few melt layers. To achieve this, rectangular NiTi samples (Fig. 3) were fabricated on AconityMINI® PBF-LB equipment using a 40-µm melt layer thickness and a bi-directional scanning strategy. To confirm the observations in the rectangular NiTi samples, NiTi cuboids (10 mm × 10 mm × 10 mm (L × W × H)) were fabricated using different hatch spacings (42 µm, 63 µm and 125 µm). The laser power, scan speed, and laser beam diameter were kept constant, to isolate the influence of the bi-directional scan behaviour from these PBF-LB processing parameters. Time series temperature data from a blackbody calibrated Kleiber KG 740—LO® pyrometer coaxially outfitted with the AconityMINI® PBF-LB equipment were used for in situ capturing of melt pool thermal radiation. The pyrometer was blackbody calibrated as installed, with a wavelength of 1200 nm, a spectral range of 1.58–1.80 μm, a measurable temperature range of 500 to 2500 °C, a response time of T90 = 10 μs, repetition (frame) rate of 100 kHz, a maximum resolution of 10 Bit, and ∅ 0.5 mm measurable field-of-view. The time series temporal data from the pyrometers are in the form of an infrared (IR) signal in the mV unit, and the x, and y coordinates of the sample on the build substrate in mm. The file size of the IR output data is approximately 44 GB, representing approximately 1 million data points per melt (build) layer. The data were post-processed with an in-house script written in Python [53], combining NumPy [54] and pandas [55] libraries. SciPy [56] was utilized for layer-by-layer statistical analysis of the data. 2D and 3D thermograms (heat maps) with ‘plasma’ and ‘turbo’ color meshes, respectively, were then constructed using Matplotlib [57] and Plotly [58]. The intensities of the respective color schemes of the reconstructed thermograms from these IR signals are representative of melt pool temperatures. Due to the difficulty in actual pyrometer temperature calibration specifically for NiTi alloy in degrees Celsius or Kelvin, and as a standard practice [59], the melt pool temperatures are reported as normalized mV values between 0 and 1.

## Results and Discussion

### Effects of Different Bi-directional Scan Patterns on the Magnitude and von Mises Stress Components

**Fig. 4:**
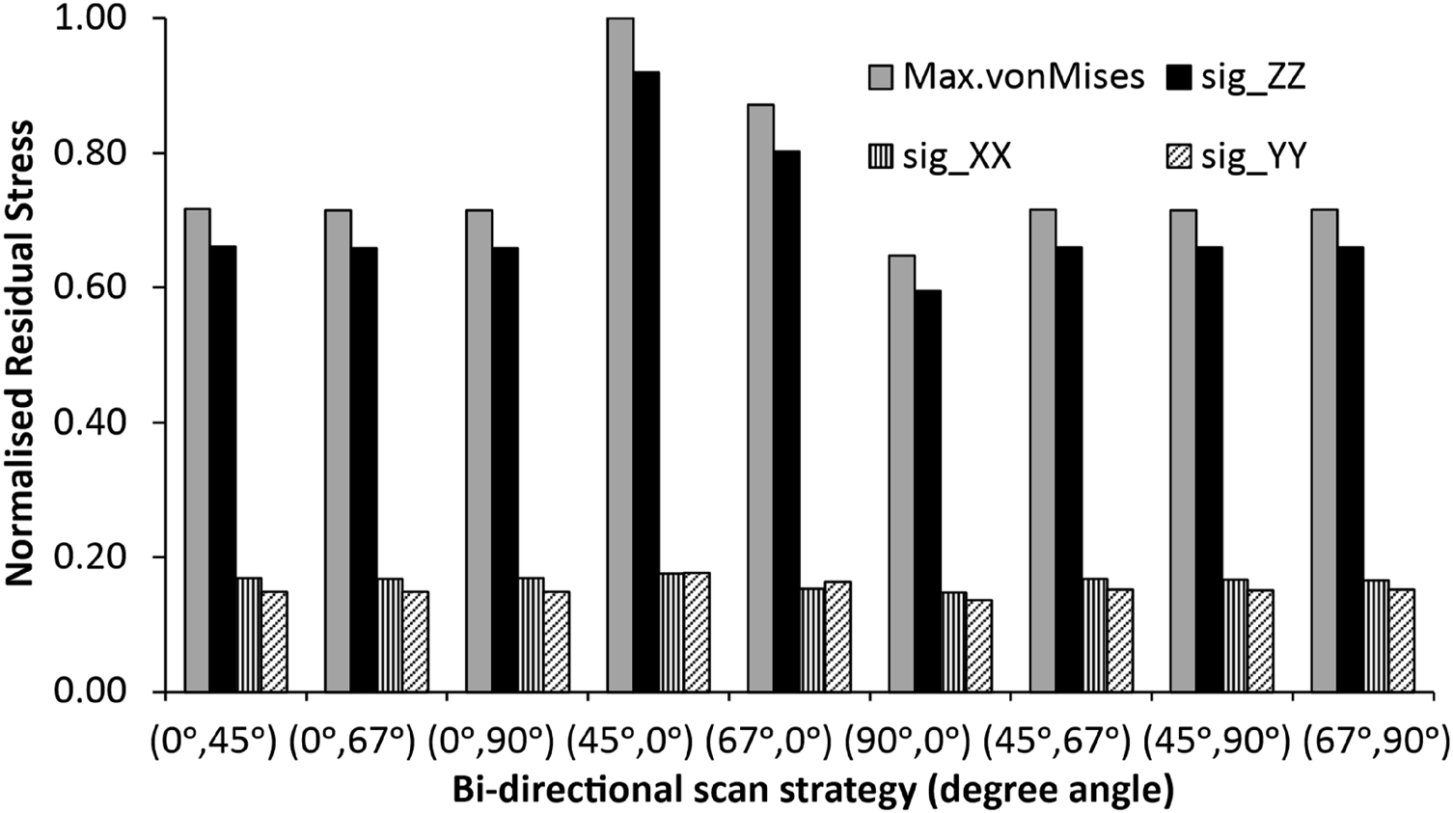
Effects of the different bi-directional scan patterns on the directional residual stress components, with part still attached to the build substrate. *X*-axis corresponds to bi-directional scan patterns No. 1 (0°, 45°), No. 2 (0°, 67°), No. 3 (0°, 90°), No. 4 (45°, 0°), No. 5 (67°, 0°), No. 6 (90°, 0°), No. 7 (45°, 67°), No. 8 (45°, 90°), and No. 9 (67°, 90°), respectively

Figure 4 shows the predicted normal residual stress components in the rectangular NiTi, resulting from the nine different bi-directional scanning patterns. Note that sig_ZZ represents the residual stress tensor component in the *Z* (build) direction, likewise for sig_YY in the *Y*-direction, and sig_XX in the *X*-direction (axis directions indicated in Fig. 3a). The predicted magnitude of von Mises stress was the lowest for No6 (90°, 0°) and highest for the No4 (45°, 0°) bi-directional scan patterns. This can be explained by the fact that large longitudinal stresses are generated parallel to a laser scan vector (laser irradiation path/direction), increasing with scan vector length, and its magnitude in a bulk component increases as the number of layers increases (part size increment) [5, 26, 60]. The evolution of sig_ZZ as a function of specimen height is provided later in this study. For No6, its start layer angle of 90° and no layer rotation for subsequent layers (i.e. 90°, 0°) implies that the laser scan vector is perpendicular to the *X*-axis (length = 55 mm) and thus has the shortest scan vector length when compared to those of No4 (45°, 0°) and No5 (67°, 0°). This scan vector length is equivalent to the width of the rectangular bar (i.e. *Y*-axis = 10 mm). Note that for all the bi-directional scan patterns, the scan vector length rarely extends the entire longitudinal length (*X* = 55 mm), except for a few layers in the No1 (0°, 45°) and No3 (0°, 90°) BDSP’s. Hence, the longitudinal stresses along the laser path rarely follow along the full length (*X* = 55 mm). Note that the maximum von Mises stress follows the same trend as the sig_ZZ von Mises presented in Fig. 4.

From Fig. 4, it can be observed that no significant differences were observed in the predicted magnitudes of residual stress between BDSPs with continuous laser scan vector rotation of 45° (No1), 67° (No2), or 90° (No3) without a prior start angle (i.e. 0°), and BDSP’s with initial start layer angles of either by 45° (No7 and 8) or 67° (No9), respectively. Furthermore, residual stresses parallel to the build direction (i.e. sig_ZZ) are the dominant residual stress component, being significantly higher than the residual stress tensor components in both X- and Y-directions, regardless of the combination of the start layer angle and layer rotation angles used. Using neutron diffraction and the contour methods, Rangaswamy et al. [61] reported similar residual stress dominance along with the build (*Z*) direction in thin walls, rectangular plates (25 mm × 5 mm × 100 mm), and square pillar (13.5 mm × 13.5 mm × 45 mm) geometries fabricated from 316 stainless steel and Inconel 718. The samples were processed using three BDSPs that included No6 (90°, 0°) in this study, and a layer rotation angle of 105° (i.e. 0°, 105°). Similarly, Vrancken et al. [39] observed that residual stresses inside PBF-LB processed Ti_6_Al_4_V compact tension specimens are mainly oriented along the build direction.

It is worth noting that this observation contrasts with the published literature for single-track analysis, in that the magnitude of von Mises stresses in the *X*-direction (sig_XX) often dominates for both unidirectional and bi-directional scan patterns [26]. From a material science perspective, this discrepancy can be explained by the competing dominance of the temperature gradient mechanism (TGM) and the cooling phase and shrinkage mechanism (CPSM) of residual stress formation in multi-layer (bulk) PBF-LB processed NiTi components. Localized TGM results from the large thermal gradients that occur around the laser spot. In this mechanism, the rapid heating of the laser track by the laser beam combined with the slow heat conduction of the solidified layer and surrounding powder particles gives rise to a steep localized temperature variation in single-track PBF-LB studies and extends into the first few layers of a multi-layer ‘bulk’ component [62]. Hence, temperature gradients develop along the laser scan length (*X*-axis), compared to both *Y-* and *Z*-directions. This results in work or strain hardening [63], and the ensuing dominance of residual stresses in the *X*-direction (sig_XX) in the few-micron layer and/or single-track PBF-LB studies. It is important to note that TGM does not require the material to be in a molten state [60]. By implication, and from a numerical analytical perspective, the melt pools that constitute the laser scan tracks are typically modelled as voxelated hexahedral elements as earlier stated in “Voxelization” section. Depending on position/location, these elements will be constrained on one to five sides and plastic strain is applied sequentially. The computed strain within the elements along the single tracks constrained by the build substrate and the ensuing anisotropic stress distribution in few-micron layer components will impact the predicted residual stress in the *X*-direction (sig_XX) than in the *Z*-direction.

However, from a numerical modelling perspective, the residual stress of a new layer is considered to be equal to the yield strength, ${\sigma }_{yield}$, since shrinkage strain (as metal shrinks on cooling from the melting point) is greater than the elastic strain [64]. The addition of new (lumped) layers increases stress distribution in the deposition direction (i.e. *Z*-direction) as moderated by material properties, SSF and ASCs; and resolved by equilibrium equations of force and moment for each voxelated element. This numerically depicts the second mechanism, CPSM. Initially, the residual stresses at the top region of the part are made up of large tensile stresses close or equal to the part’s yield strength, ${\upsigma }_{\text{yield}}$. By extension, and from a material science perspective residual stresses form during the cool-down phase of the molten top layers. The cooling molten top layers shrink due to thermal contraction, but it is inhibited by the underlying material. The underlying material is typically the substrate in single-track experiments or the prior solidified layer in a multi-layer ‘bulk’ part which may have been partly remelted. This early stage shrinkage inhibition of a few layers is characterized by tensile stresses on the top side of the solidified track and compressive stress below [60, 65]. Considering the overall bulk component, an increase in the distance between the substrate and topmost layer results in a compressive topmost layer and layers close to the substrate in a tensile stress state. This has been experimentally demonstrated in the studies by Refs [39, 60], and of this study, evidence of this is presented in the succeeding sections. The overall magnitude of residual stresses increases along the build height (*Z*-direction) as the stress imbalances increases due to CPSM. Hence, why the magnitude of von Mises stress in Fig. 4 follows the sig_ZZ. Note that the term compressive stress in the context of residual stress phenomena refers to sections or regions at low von Mises stress states, while the term, tensile stresses refer to sections or regions at higher von Mises stress states. This contrasts with classical mechanistic compressive stresses which are denoted by a stress value with a negative sign (i.e. –ve MPa).

Although the stress values in this study were normalized and qualitative, the general trend of the predicted residual stress location and/or distribution remains fairly unchanged [23, 34, 35].

### Contribution of the Bi-directional Scan Patterns (BDSP) to Anisotropic Stress Distribution

**Fig. 5:**
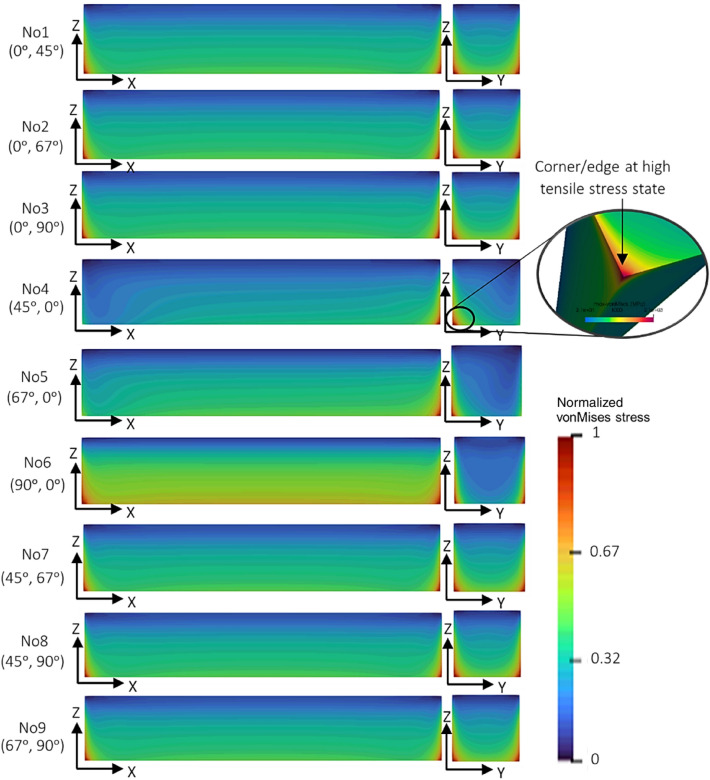
Residual stress contours of NiTi rectangular bars simulated with bi-directional scan patterns (BDSP) with 9 different combinations of start layer scan vector angle and subsequent layer scan vector rotation angles. Note that the scan vectors refer to hatching lines which guide the laser path. More so, for all cases, the simulated component is still attached to the substrate, and the topmost sections at maximum build height are in compressive stress states according to the normalized von Mises color bar

The residual stress contours in Fig. 5 reveal the von Mises stress distribution within the NiTi rectangular bar, due to the different BDSPs. As with Fig. 4, there is not much difference in the contour profile of No1 (0°, 45°), No2 (0°, 67°), and No3 (0°, 90°) in Fig. 5. This implies that the differences between the residual stress development and distribution in the rectangular bar are little to-negligible when the angle of the laser scan vectors for subsequent layers is rotated by either 45°, 67° or 90°. In contrast, large differences in the residual stress distribution are observed, when the laser scan vectors for subsequent layers are kept constant at either 45°, 67° or 90° (i.e. No4, No5, or No6 in Fig. 5) throughout the build height. It is worth noting that the scan vector rotation angle plays a major role in the surface roughness, density and microstructure of the finished part [12, 26].

**Fig. 6:**
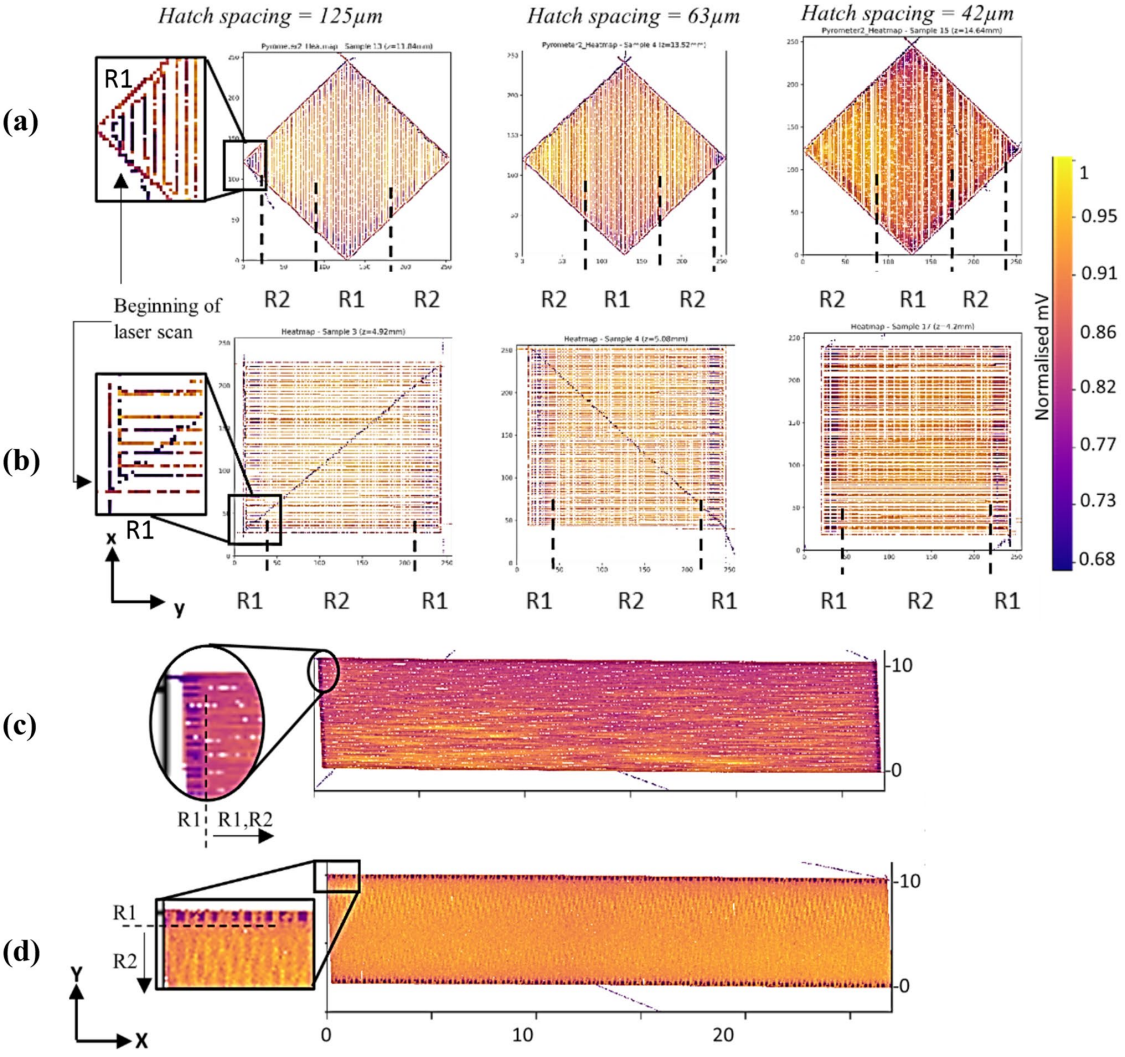
Reconstructed 2D thermograms (representative single layer heat maps) of; **a** three different NiTi cubes fabricated using bi-directional scan patterns with the laser scan vectors traversing diagonally (diagonal laser hatching—No. 4 (45°, 0°)), **b** parallel to *Y*-axis (bi-directional scan No. 6 (90°, 0°)), **c** NiTi rectangular bars, fabricated using bi-directional scan patterns with the laser scan vectors parallel to the *X*-axis, and **d** parallel to the *Y*-axis (i.e. No. 6 (90°, 0°) of this study). R1 and R2 represent Regions 1 and 2 at low and high spatial temperatures, respectively, and the thermograms were reconstructed from in situ melt pool thermal radiation signals during fabrication. In the case of NiTi cuboids, three different hatch spacings (125 µm, 63 µm and 42 µm) were used, whilst all other parameters were kept constant

**Fig. 7:**
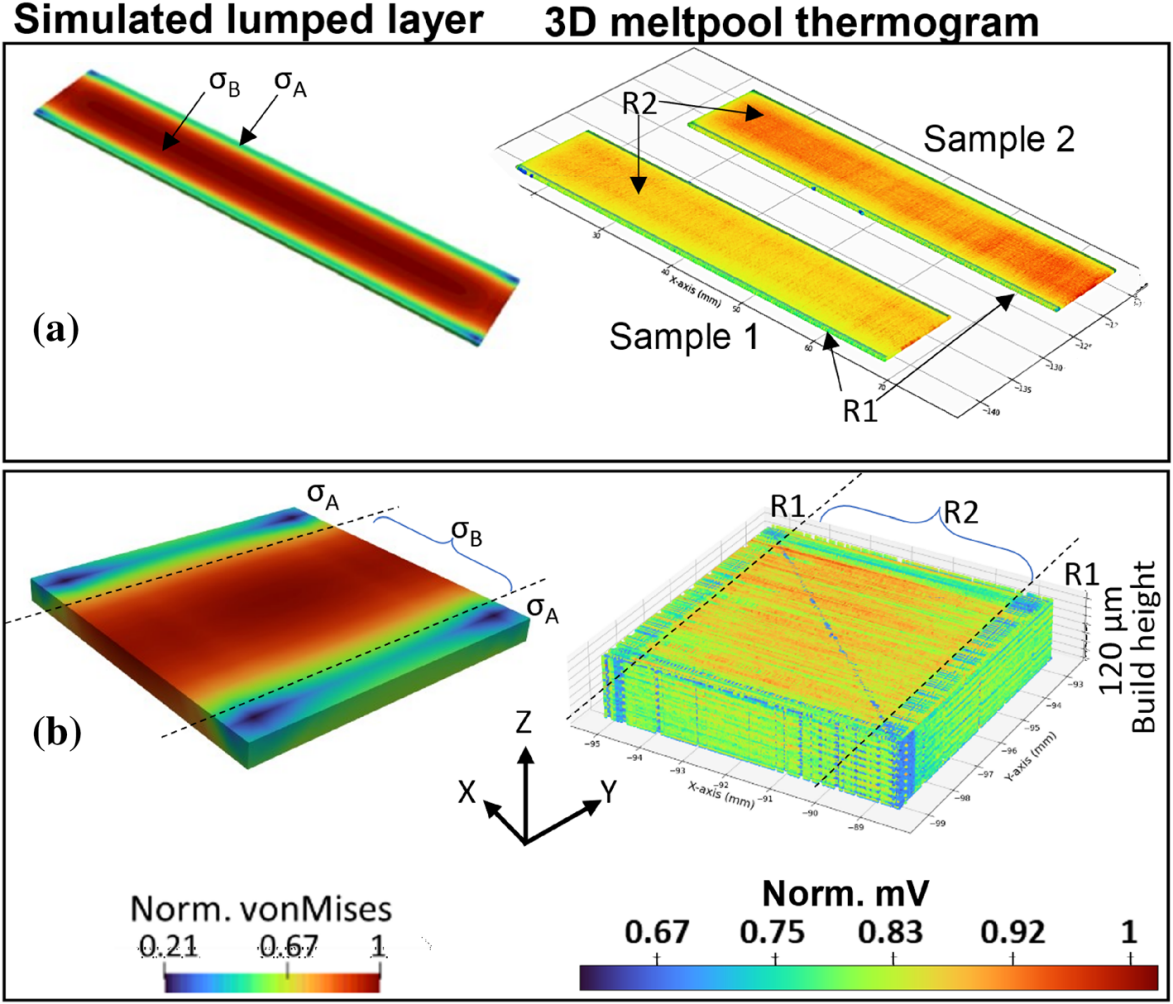
von Mises stress distribution of first simulated lumped layer, and corresponding 3D thermograms reconstructed from in situ melt pool thermal radiation data for; **a** two rectangular NiTi samples processed at different parameters but with same bi-directional laser scan pattern, No. 6 (90°, 0°), and **b** first 120 µm build height of cubic NiTi samples fabricated with bi-directional laser scan patterns No. 6 (90°, 0°). Note that *σ*_*A*_ and *σ*_*B*_ represent areas of low and high von Mises stress, respectively, with corresponding regions of low and high spatial temperatures, R1 and R2

From Fig. 5, the locations of maximum residual stresses are at the corners of the rectangular bars, and close to the build substrate. This is consistent with the observations by Marques et al. [15] in PBF-LB processed Ti–6Al–4V assessed via coupled 3D thermomechanical modelling and the study by Ahmad et al. [66] in PBF-LB processed Ti–6Al–4V and Inconel 718, assessed via inherent strain-based numerical simulation and experimentally validated with the contour method. The study by Lua et al. [32] on the residual stress formation of a Ti–6Al–4V part fabricated by the DED process also demonstrates the location of maximum stress at the part–substrate interface. It is important to note that irrespective of the differences in spot sizes and the scan speeds, DED and PBF-LB processes both use metal powders of a similar particle size range (< 100 µm), produce similar peak temperatures between 2000 and 2500 K, have similar high cooling rates between 10^4^ and 10^6^ K/s, similar thermal gradients in the order of ~ 5 × 10^4^ K/cm, and fabricate components with similar residual stress levels [67]. For BDSP No4 (45°, 0°) in Fig. 5, the location of the highest stress concentration is at diagonally opposite corners of the rectangular bar, close to the build substrate. This is partly explained by the temperature gradient mechanism (TGM) for residual stress formation (refer to section  "Effects of Different Bi-directional Scan Patterns on the Magnitude and von Mises Stress Components") and substantiated by 2D and 3D thermograms (heat maps) in Figs. 6 and 7. Although isotropic inherent strain model of the scan pattern simulation does not consider thermal effects, a remarkable similarity in the predicted stress locations and melt pool thermal radiation profile of first few layers (< 500 µm) during actual PBF-LB processing of NiTi was observed.

Existing studies on quadrilateral geometries (i.e. rectangular bars, rectangular plates, square pillars, regular cuboids) have all been reported to display the highest residual stress concentrations at corners [32, 68], regardless of the material used. To visually explain the temperature gradient mechanism (TGM) for residual stress formation in the rectangular bars, reconstructed 2D layer heat maps (thermograms) of 10 mm × 10 mm × 10 mm NiTi cuboids and 55 mm × 10 mm × 10 mm NiTi rectangular bars are presented in Fig. 6. Note that the axis coordinate for the cuboid in Fig. 6a, b has been adapted to match the axis coordinates for the rectangular bar in Fig. 6c, d, where *Y*-axis represents the width (10 mm) and the length is *X*-axis (55 mm). In Fig. 6a, the laser scan vectors traverse diagonally through the cuboids (diagonal hatching) and in Fig. 6b, and the laser scan vectors are parallel to the *Y*-axis. It is important to note that the diagonal laser hatching as presented in Fig. 6a is the same as BDSP No4 (45°, 0°) in the rectangular bar. It can be observed from Fig. 6 that the corners and some edges of the layers are the locations of a less optimal laser activity, due to the ramp-up and ramp-down sinusoidal wave modulation response of the laser during the beginning and end of scan vector lengths [69].

Specifically, at these locations, the laser ramps up to power and speed at the beginning of the track, travels a relatively short distance at full power and speed (steady-state), and then the slowing down of the laser at end of the track (ramp-down), making the turn for the start of a new track in the opposite direction (see the magnified 2D thermograms for NiTi hatch spacing = 125 µm in Fig. 6a, b). This creates spatial temperature regions marked as; R1 for the low spatial temperature region and R2 for the high spatial temperature regions in Fig. 6. In the case of BDSP No4 (45°, 0°) (i.e., diagonal laser hatching) in Fig. 6a, it shows that the diagonal corners of the NiTi cube have unique thermal temperature distributions; (1) the corner which marks the beginning of the laser scan being at lower temperatures (R1), (2) a transitioning to R2, where the scan vector length and inter-track laser scan time is short, (3) vertical mid-sections at R1 due to longer scan vector lengths and longer inter-track laser scan times, and (4) diagonally opposite corner at R2 marking the end of the laser pass for that layer. Also note the predominance of low spatial temperature areas (i.e. R1) across the mid-section of the rectangular bar in Fig. 6c. With layer scan vectors which are parallel to the *X*-axis (*X* = 55 mm) in Fig. 6c, the scan vector lengths and inter-track laser scan times are greatly amplified (i.e. R1 regions) when compared to the vertical mid-sections of the cuboid in Fig. 6a.

For BDSP No6 (90°, 0°) in Fig. 6b, d, the locations of less optimal laser activity coincide with the regions at low spatial temperatures (R1) and are aligned parallel to Y-axis (Y = 10 mm), while the mid-sections are at higher spatial temperatures (R2). Following the concept of temperature gradient mechanism (TGM) for residual stress formation in the planar 2D layer, these locations at lower spatial temperature (i.e. R1) infer locations at lower residual tensile stresses (*σ*_*A*_), while the locations at higher spatial temperature (i.e. R2) experience higher residual stress (*σ*_*B*_). This agrees with the observations by Ref. [7, 26], in that residual stress decreases towards the end of a track (identical to locations of a less optimal laser activity in this study), due to lower temperature gradients (R1 = *σ*_*A*_), and conversely, R2 = *σ*_*B*_. More so, according to Buchbinder et al. [5], maximum plastic compression (lower relative von Mises stress, *σ*_*A*_) forms at the beginning of a scan vector. As an illustrative example, Fig. 7a, b shows the von Mises stress contour of the first simulated lumped layer, and corresponding 3D thermograms reconstructed from collated *in situ* melt pool thermal radiation data of a few layers of PBF-LB processed NiTi samples processed using BDSP No6 (90°, 0°). Note that the simulation set-up for the cubic NiTi shown in Fig. 7b follows exactly the same methodology described in “Methodology” section. Being that there is no layer scan rotation in both BDSP No4 (45°, 0°), No5 (67°, 0°), and No6 (90°, 0°), the laser scan tracks (and the solidified tracks) are stacked in the exact coordinates throughout the build height. However, it is important to note that the melt pool radiation data are localized instantaneous time series data, which is incapable of qualitatively (pictorially) capturing the global effect of heat accumulation and thermal gradient as build height increases. In the context of this study, the presented thermograms are only suitable in explaining the TGM of residual stress formation in single layers and few micron-layer components. However, the thermal gradients and ensuing thermally induced residual stresses across the build height in PBF-LB processed ‘bulk’ NiTi can be deduced by in-depth quantitative (statistical) analysis of the collated in situ melt pool thermal radiation data, as demonstrated by Monu et al. [70].

Meanwhile, there are no significant differences in the overall magnitude and distribution of the residual stresses between BDSP No3 (0°, 90°) and No8 (45°, 90°). These two BDSPs are popularly used in the literature and the relevance of having an initial start layer angle of 45° is mainly to match the orientation of part/component on a build substrate [71]. If the part is oriented at 45° to the direction of the re-coater blade motion, then a start layer angle rotation of 45° is required to: (1) reduce powder re-coater interference to the solidifying last layer, (2) allow the solidifying tracks to be always parallel to one side of the sample, and (3) to avoid over-or under-heating at the corners.

### Residual Stress Evolution with the Increase in Build Height

**Fig. 8:**
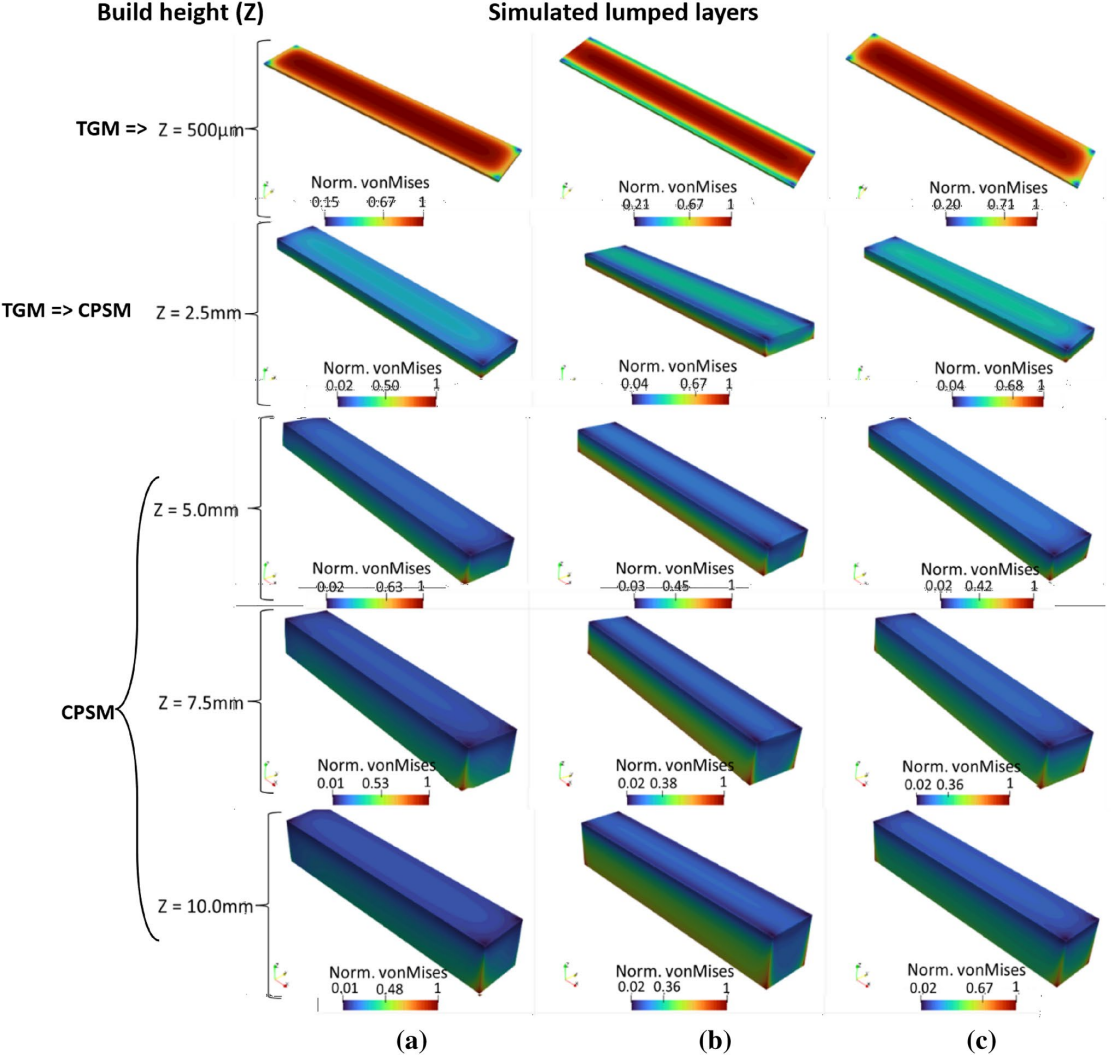
Evolution of stress field and the corresponding residual stress formation mechanism regimes for every increase in simulated lumped layers (i.e., build height) using bi-directional scans **a** No. 4 (45°, 0°), **b** No. 6 (90°, 0°), and **c** No. 8 (45°, 90°). TGM and CPSM represent the temperature gradient mechanism and cooling phase shrinkage mechanism, respectively. Norm. von Mises represents normalized von Mises

The evolution of residual stresses in the NiTi rectangular bars using BDSP No4 (45°, 0°), No6 (90°, 0°) and No8 (45°, 90°) is presented in Fig. 8. For the sake of brevity, further analysis will be focused on these three bi-directional scan patterns (BDSPs) for the following reasons: from the preceding sections, BDSP No4 (45°, 0°) and No6 (90°, 0°) resulted in the highest and lowest magnitudes of residual stresses, respectively, and No8 (45°, 90°), for its popularity amongst researchers and with negligible qualitative differences in the predicted distribution and magnitude of residual stresses compared to other BDSPs with layer scan rotations.

The addition of new layers requires full melting of the new powder particles and solidification. Hence, residual stress formation in the bulk NiTi part via TGM (as discussed in “Contribution of the Bi-directional Scan Patterns (BDSP) to Anisotropic Stress Distribution” section) is exasperated by the cooling phase and shrinkage mechanism (CPSM) as the build height increases. Figure 8 shows distinct stress distribution profiles after the deposition of lumped layer thicknesses of 0.50, 2.5, 5.0, 7.5 and 10.00 mm (maximum build height). At 0.50 mm build height (Fig. 8a), the solidified NiTi parts are mainly in a tensile (i.e. higher von Mises) stress state. However, initial part distortion is constrained by its attachment to the substrate. Note that reduction in distortion by the substrate does not dissipate the bending forces; instead of causing distortion, it results in higher locked-in residual stresses [72]. The distribution of the compressive (i.e. lower von Mises) stresses corresponds to the sections at a lesser thermal activity as already discussed. This is mainly evident in the No6 (90°, 0°) bi-direction scan (see Fig. 8b at *Z* = 500 µm i.e. 05 mm) where the lower stress sections (*σ*_*A*_) correspond to the beginning and end of the laser scan vectors which are oriented at 90° to the *X*-axis (refer to the thermograms in both Figs. 6 and 7).

**Fig. 9:**
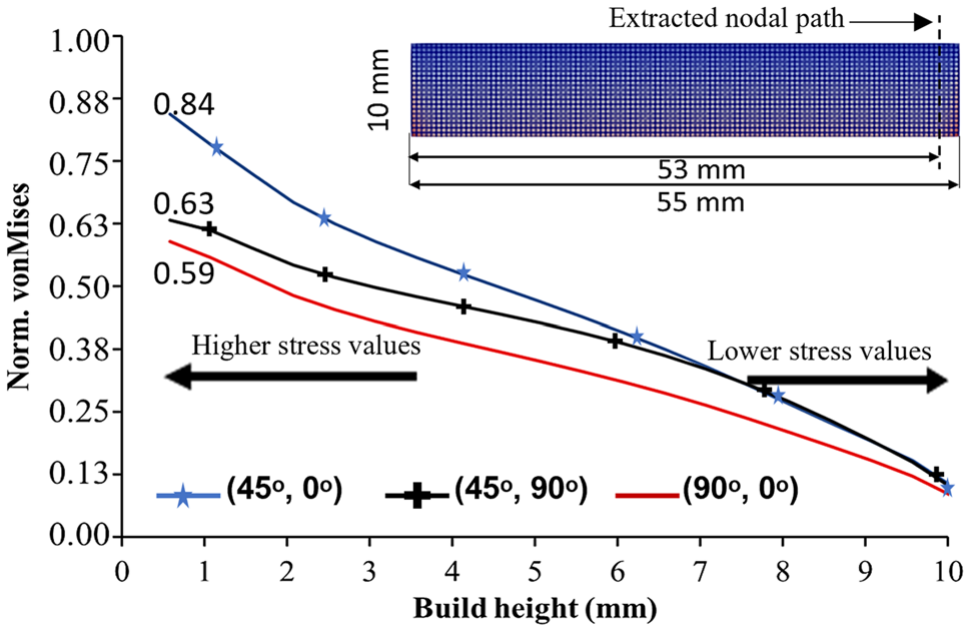
The von Mises stress evolution with change in build height (*Z* = 0.5–10 mm), extracted at nodal points 2 mm from the edge along the longitudinal section (*X*-axis)

A shift from a predominantly high (i.e., tensile) residual stress state at 500 µm build height, to a relatively lower (i.e., compressive) residual stress state is observed from 2.5 mm build height. This suggests a relative homogeneity in the temperature field of the substrate and the solidified layers and the ensuing reduction of the thermal gradient. The studies by Refs. [32, 70, 71] have demonstrated that indeed, a few micron layers from the build substrate is required to attain homogeneous temperature distribution during PBF-LB processing of NiTi parts/components. This typifies the onset of the cooling phase and shrinkage mechanism (CPSM) predominance over TGM, in the formation of residual stresses as build height increases. Continuing the PBF-LB process (the simulated lumped layer from *Z* = 5 mm to 10 mm in Fig. 8), the cooling and shrinkage of the different layers provoke a gradual increment in the compressive stresses at the towards to topmost sections of the components along the build height. When the deposition/simulation of all the layers is completed at *Z* = 10 mm, the material solidifies, the bulk rectangular bar begins to cool, and the topmost surface is exposed to the relatively cooler build chamber atmosphere and surrounding powder particles. The thermal contraction at the top of the part stretches the lower portions of the component so that it bends upward (bowling). This thermal contraction of the upper layers results in the mechanical compression of the upper surface of the rectangular bar, while the bottom surface and relevant corners are subjected to tensile stresses [72]. A similar trend in rectangular and S-shaped Ti–6Al–4V parts has been reported by Lu et al. [32]. In double cantilever beams, the largest stresses were also reported by Cheng et al. [68] to be at the four bottom corners of the beam and the bottom surface of the centre pillar. Note that these corners in contact with the substrate are features of quadrilateral geometries that constitute locations at the highest state of tri-axial stresses between the part and the substrate. Therefore, lower levels of stress, i.e. lower von Mises stress levels, are observed as build height increases (i.e. greater distance away from the tri-axial stress points/locations or as observed in the study by Rangaswamy et al. [61], residual stresses decrease strongly as the free end is approached). As shown in Fig. 9, this trend is portrayed in the current study, in that the predicted von Mises stresses decreased from starting normalized values of 0.84, 0.63, and 0.59, at the build substrate surface (*Z* = o mm) to below 0.13 at the topmost build surface (*Z* = 10 mm) for the BDSP No4 (45°, 0°), No6 (90°, 0°) and No8 (45°, 90°), respectively. Specifically, in the study by Watkins et al. [63], the residual stress profile extracted from a similar nodal location/path (70 mm in their case) corroborates with the residual stress trend in Fig. 9. Both locations (53 mm in our case, and 70 mm in Watkins et al. [63]) are at the end of laser passes (far right-hand side of the beam/bar) in typical PBF-LB processing, as controlled by the laser scan vector (hatch line) coordinate system (refer to Fig. 2b). More so, both locations are the closest to the bar corners where the tri-axial stress states are highest as discussed in “Contribution of the Bi-directional Scan Patterns (BDSP) to Anisotropic Stress Distribution” section. This explains why most component delamination after PBF-LB processing occurs at the component–substrate interface, starting from these bar corners/edges at the highest stress state (i.e. tensile stress conditions).

### Magnitude of Distortion

**Fig. 10:**
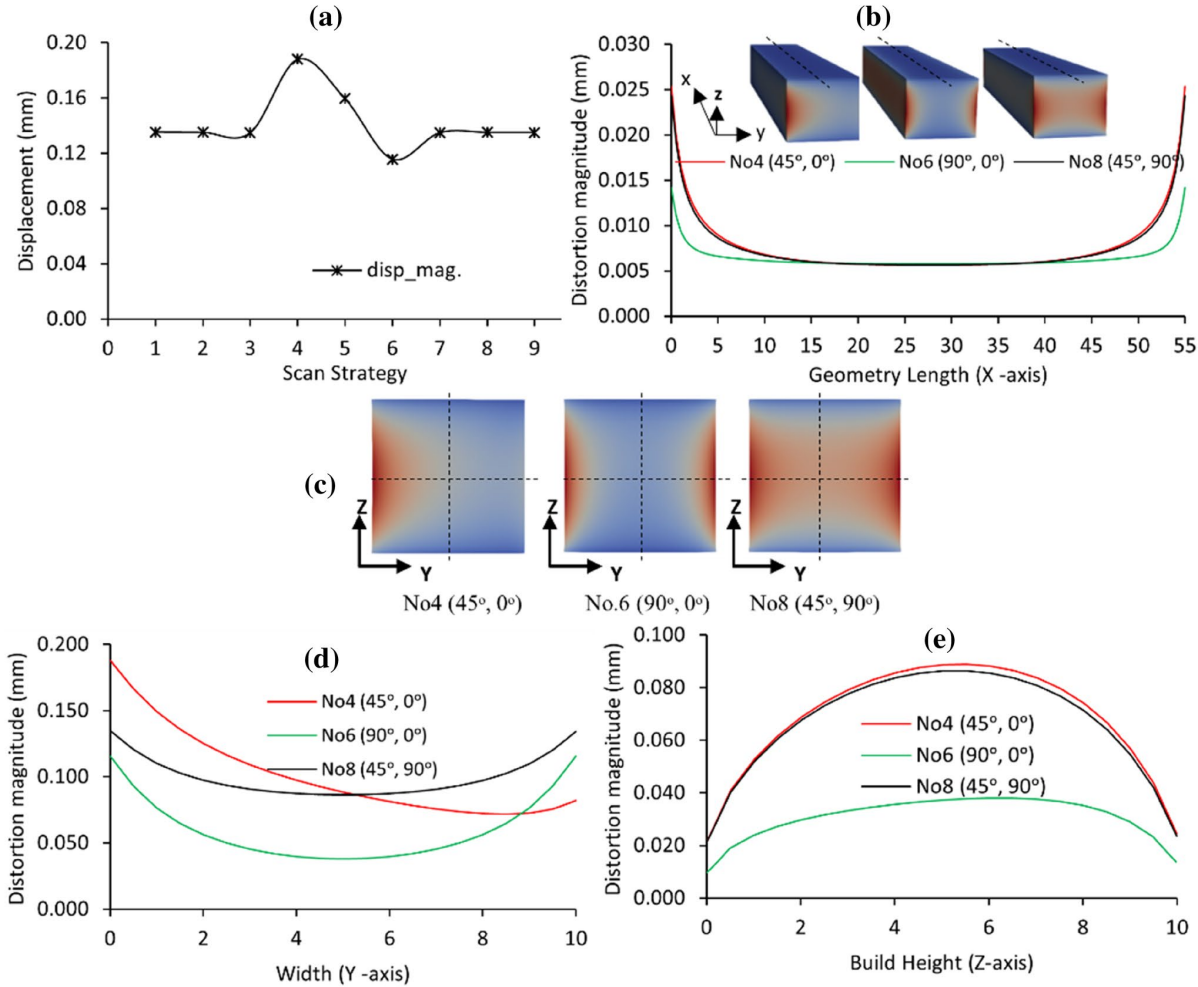
Effects of the different bi-directional scan patterns on the **a** overall magnitude of displacement, **b** predicted displacement along the *X*-axis, **c** distortion contours and nodal path locations, **d** predicted displacement along the *Y*-axis, and **e** *Z*-axis, with the rectangular bar still attached to the build substrate (note that the build substrate is not shown)

A graphical plot of the simulated magnitude of displacement and individual distortion values along with the *X*-, *Y*- and *Z*-axis, due to the different BDSPs, is shown in Fig. 10. The profile of the on-plate magnitude of displacement curve in Fig. 10a matches that of the von Mises stress in Fig. 4a. This is attributed to the predisposition that components with high locked-in residual stresses generally experience relatively higher magnitudes of displacement regardless of the restraining action. The highest predicted overall magnitude of displacement is about 0.18 mm for BDSPs No4 (45°, 0°) and No6 (90°, 0°) resulting in the least magnitude of displacement (circa 0.11 mm) in the NiTi rectangular bar. From the longitudinal (nodal path along the *X*-axis) distortion curves in Fig. 10b, the NiTi rectangular bar experiences upward curving of the edges (i.e. a bowl-shape) for all three BDSPs: No4 (45°, 0°), No.6 (90°, 0°), and No8 (45°, 90°). The degree of curvature (i.e. bowling of the edges) for both No4 (45°, 0°), and No8 (45°, 90°) is higher and steeper than that observed for No6 (90°, 0°).

Figure 10c shows the distortion contours and nodal path locations of interest in the NiTi rectangular bar. Regarding the distortion curves traced from horizontal nodal paths along *Y*-axis as shown in Fig. 10c, a clear distinction is observed in Fig. 10d. The distortion curves are similar for both No.6 (90°, 0°) and No8 (45°, 90°), with the latter being at a higher magnitude. Conversely, using BDSP No4 (45°, 0°) resulted in a rectangular bar with a greater degree of distortion on the left edge/side compared to the right. It also corresponds to the diagonal corner concentration of residual stresses in the rectangular bar as previously discussed (refer to Fig. 5). According to the study by Li et al. [19], the formation of the bowl-shaped distortion can be explained by the cooling phase and shrinkage mechanism (CPSM) previously discussed in “Residual Stress Evolution with the Increase in Build Height” section. In Fig. 10d, an inverted bowl-shaped distortion curve is observed by tracing the vertical nodal paths along *Z*-axis in Fig. 10c.

## Conclusion

For the PBF-LB processing of NiTi, BDSPs No4 (45°, 0°) and No6 (90°, 0°) resulted in the highest and lowest residual stress build-up, respectively. No significant variations were observed in the predicted magnitudes of residual stress between BDSPs with continuous laser scan vector rotation of 45° (No1), 67° (No2), or 90° (No3) without a prior start angle (i.e. 0°), and BDSPs with initial start layer angles of either by 45° (No7 and 8) or 67° (No9), respectively. However, a striking similarity between the evolution of the stress field and melt pool thermal radiation during actual PBF-LB processing of Nitinol was observed. This is mainly evident in the No6 (90°, 0°) BDSP where the compressive stress sections correspond to the beginning and end of the laser scan vectors which are oriented at 90° to the *X*-axis and contributed to a higher thermal gradient. The striking similarities in the thermograms with the predicted residual stress contours are considered a novelty in this study, as it provides a deeper insight into the temperature gradient mechanism (TGM) of residual stress formation in single layers and few micron-layer components.

From the results of this study, BDSP No.6 (90°, 0°) is the best candidate for processing NiTi with reduced residual stresses and displacement. However, the persistent alignment of the laser scan tracks at 90° for all layers throughout the build height will create highly preferential grain orientations and grain growth direction. This microstructural directionality may induce extreme degrees of anisotropic mechanical behaviour [12, 73], with lower tensile strength in the longitudinal direction (X-axis) compared to the build direction (Z-axis). By extension, the compressive strength in the longitudinal direction will be higher compared to the build direction. This is also valid for NiTi rectangular bars fabricated with BDSPs No5 (67°, 0°), and No6 (90°, 0°). It has been shown that under axial tensile loads, the structural properties of such printed AM components without rotation are inferior to those fabricated with rotated laser scan vectors per layer [i.e. No1 (0°, 45°), No2 (0°, 67°), No3 (0°, 90°), No7 (45°, 67°), No8 (45°, 90°), and No9 (67°, 90°)]. This may be attributed to the relatively increased microstructural non-directionality due to the ever-so-slightly changing laser–powder interaction dynamics, melt pool solidification front and thermal gradients induced by the rotating scan vectors for every new layer. Fatigue properties could also be severely negatively affected by the anisotropy of residual stress distribution [22]. Regarding surface roughness, the staircase effect of PBF-LB will be more pronounced in components fabricated using BDSPs with laser scan rotation per layer (i.e. No4 (45°, 0°), No5 (67°, 0°), and No6 (90°, 0°).

This work provides insight towards a qualitative understanding of the effect of the bi-directional scan strategy on the trend of three-dimensional residual stresses, residual stress evolution and distortion for the PBF-LB processing of NiTi alloys. This was accomplished by using the scan pattern simulation module in Ansys Additive Print® 2021 software suite, which is based on an isotropic inherent strain numerical model. Using a bi-directional scan pattern with either a stating layer angle of 0°, or 45°, and subsequent layer rotation angles of 90° provides a reasonable compromise between residual stresses, distortion, and anisotropic mechanical/structural properties. The stress values and distortions values presented in this work are not absolute since a further calibration step for the strain scaling factor (SSF) and anisotropic strain coefficients (ASC) is required.
